# Magel2 deficiency promotes cardiac remodeling and increases arrhythmogenic susceptibility in a mouse model relevant to Prader–Willi and Schaaf–Yang syndromes

**DOI:** 10.1042/CS20260647

**Published:** 2026-06-22

**Authors:** Laura Dötsch, Leo Weirauch, Janika Hunger, Tobias Thiemann, Johann Maaß, Constanze Schmidt, Florian Leuschner, Christian P. Schaaf, Henning Fröhlich

**Affiliations:** 1 Institute of Human Genetics, Heidelberg University, Heidelberg, Germany; 2 Department of Internal Medicine III, University Hospital Heidelberg, Germany; 3 German Centre for Cardiovascular Research (DZHK), Heidelberg, Germany

**Keywords:** Concentric cardiac remodeling, Magel2, Prader-Willi syndrome, Schaaf-Yang syndrome

## Abstract

Prader–Willi syndrome (PWS) and Schaaf–Yang syndrome (SYS) share overlapping phenotypic features, but potential cardiac involvement in both conditions remains poorly understood. Here, we investigated cardiac function in *Magel2* knockout (KO) mice, a model relevant to PWS and SYS, to assess the impact of Magel2 deficiency on the heart. Echocardiographic analysis of 20-week-old *Magel2*-KO mice revealed concentric remodeling of the left ventricle together with reduced left ventricular end-diastolic volume and stroke volume, as well as a modest but significant reduction in ejection fraction. Electrophysiological studies identified sex-dependent alterations, particularly in males, characterized by shortened action potential duration and increased atrial potassium currents. Surface electrocardiography recordings showed no overt arrhythmias and visual inspection confirmed sinus rhythm during the monitoring period; however, the observed cellular alterations indicate increased arrhythmogenic susceptibility. In addition, aged *Magel2*-KO mice developed adult-onset obesity and exhibited elevated HbA1c levels consistent with impaired glycemic control. Given the minimal expression of Magel2 in cardiac tissue, these findings suggest that systemic and metabolic alterations may contribute to the observed cardiac phenotype. Together, these results demonstrate structural cardiac remodeling and electrophysiological changes consistent with increased arrhythmogenic susceptibility in Magel2-deficient mice, suggesting clinically relevant cardiac involvement in SYS and PWS. Our findings support consideration of structured cardiovascular monitoring to mitigate potential secondary cardiac complications in affected individuals.

## Introduction

Prader–Willi syndrome (PWS [MIM: 176270]) results from pathogenic alterations in the maternally imprinted PWS region on chromosome 15q11–q13. These alterations include paternal deletions of a 5–6 Mb segment, maternal uniparental disomy of chromosome 15, or imprinting defects affecting the regulatory region [1]. In contrast, Schaaf–Yang syndrome (SYS [MIM: 615547])—a genetically and clinically related disorder—is caused by truncating mutations in *MAGEL2*, a gene located within the same chromosomal region [2,3]. Both syndromes share a range of phenotypic features [4].

In infancy, affected individuals commonly present with hypotonia, feeding difficulties, and respiratory problems requiring intensive medical support [3]. As they age, motor impairments, intellectual disability, and behavioral abnormalities become more apparent [3,5]. A distinguishing feature of SYS is the presence of joint contractures, particularly affecting distal joints, which may result in arthrogryposis multiplex congenita in severe cases [3]. Additionally, up to 80% of individuals with SYS meet diagnostic criteria for autism spectrum disorder—substantially more than in PWS [3]. While hyperphagia and morbid obesity typically emerge around age eight in PWS, similar eating behaviors may also occur in SYS, albeit usually later in life [6].

Beyond the well-characterized neurodevelopmental features, emerging evidence suggests that the cardiovascular system may also be involved in both disorders. Cardiovascular disease and heart failure are the second leading causes of death in individuals with PWS [7,8], and congenital heart defects have been reported in case studies of individuals with SYS [9,10]. Moreover, alterations in cardiac autonomic regulation and electrocardiographic parameters have been described in children with PWS, indicating that functional cardiac abnormalities may manifest early. However, the underlying mechanisms of these cardiovascular manifestations remain largely unknown.

Despite these clinical observations, no studies to date have systematically examined the cardiac consequences of Magel2 deficiency. In the present study, we used *Magel2* knockout (KO) mice as a model of Magel2 deficiency relevant to SYS and PWS to investigate the impact of Magel2 loss on cardiac structure and function. Our findings offer novel insights into the pathophysiology of SYS and potentially PWS, and may inform future strategies for early cardiovascular risk assessment and intervention.

## Material and methods

### Animals

Mice were housed in the Interfaculty Biomedical Facility at Heidelberg University, Germany, under specific pathogen-free conditions with a 12-hour light/dark cycle and *ad libitum* access to food and water. No anaesthetics were required for the procedures performed. Mice were killed by CO_2_ inhalation using a gradual fill method (approximately 20%–30% chamber volume per minute), and neonatal mice up to postnatal day 12 were killed by decapitation, in accordance with institutional and regulatory guidelines. All animal procedures were approved by the Regierungspräsidium Karlsruhe, Germany, and conducted in accordance with the German Animal Welfare Act (Tierschutzgesetz).

### Generation of *Magel2*-KO animals

C57BL/6-*Magel2*^tm1Stw^ mice [11] were purchased from Jackson Laboratory (Bar Harbor, Maine, USA). To obtain mice deficient of Magel2 (*Magel2*^m+/p-^, from here on *Magel2*-KO mice), functional wild-type (WT) males carrying the LacZ-knock-in-construct replacing *Magel2* on the maternal allele (*Magel2*^m-/p+^) were crossed with female C57BL/6-WT. Animal studies were approved by the Regierungspräsidium Karlsruhe, Germany (approval number: G-252/19, G-299/19).

### RNA isolation and cDNA synthesis

Total RNA was prepared from frozen mouse heart tissue samples using TRIzol reagent (Thermo Fisher Scientific, Waltham, MA, USA). First strand cDNA synthesis was performed from 1.5 μg of total RNA using a Superscript II reverse transcriptase kit (ThermoFisher Scientific, Waltham, MA, USA) and oligo dT12-18-primers (ThermoFisher Scientific, Waltham, MA, USA) according to the manufacturer’s instructions.

### Quantitative real-time PCR

Quantitative real-time PCR was performed using QuantStudio Real-Time PCR system with an annealing temperature of 60°C using qPCRbio Sygreen Blue Mix Lo ROX (PCR Biosystems, London, UK) according to manufacturer’s instructions. Each of the samples was analyzed in triplicate and relative mRNA levels were assessed using the Standard Curve Method by normalization to succinate dehydrogenase complex subunit A (*Sdha1*) and hypoxanthine phosphoribosyltransferase 1 (*Hprt1*). All primer sequences are listed in Supplementary Table S1.

### HbA1c quantification

For HbA1c determination, 20 μl of whole blood were homogenized in 80 μl of ice-cold deionized water, incubated on ice for 10 minutes, and centrifuged at 15,000 ***g*** for 10 minutes at 4°C. Ten microliters of the resulting supernatant were transferred to HPLC vials containing 125 μl of 20 mM Bis–Tris and 2 mM KCN (pH 6.9). The HbA1c fraction was analyzed using HPLC with a PolyCAT A column (35 mm × 4.6 mm, 3 μm, 1500 Å) from PolyLC Inc. (USA) on a LaChrom Elite HPLC system from Hitachi High-Technologies Corporation (Japan). Identification of the HbA1c fraction was performed by comparing its retention time with that of a pure hemoglobin standard. The HbA1c content was quantified as a percentage of non-glycated hemoglobin by calculating the ratio of the peak areas of HbA1c and Hb0.

### Echocardiography

Echocardiography was performed during the active (dark) phase using the FUJIFILM VisualSonics Vevo 2100 Imaging System (FUJIFILM VisualSonics, Inc., Toronto, Canada) equipped with an MS-550D Micro Scan transducer. Left parasternal long-axis views were visualized in B-mode to calculate ejection fraction, end-diastolic volume, and end-systolic volume. Anterior and posterior wall thickness were measured in M-mode in a short axis view. All analyses were conducted using VevoLab software. Both image acquisition and data analysis were performed by a blinded investigator.

### Electrocardiography

Electrocardiographies (ECGs) were carried out using the ecgTUNNEL system (emka technology) after a five-minute acclimatization period of the animals in the tunnel. Data evaluation was carried out via cgAVG module software.

### Electrophysiological analysis

After removal mouse hearts were immediately placed in Ca^2+^-free Tyrode’s solution (30 mM BDM, 100 mM NaCl, 10 mM KCl, 1.2 mM KH2PO4, 5 mM MgSO4, 50 mM Taurine, 5 mM MOPS, 20 mM Glucose; pH adjusted to 7.0) for a maximum duration of 15 minutes. Electrophysiological measurements were performed as reported earlier [12,13]. Electrophysiological recordings were focused on atrial cardiomyocytes, as TASK-1 (K2P3.1) channels are predominantly expressed in atrial tissue and play an established role in atrial electrophysiology and atrial fibrillation (AF). Briefly, atria were dissected and rinsed in Ca^2+^-free Tyrode’s solution. Tissue was digested using collagenase type I (288 U/ml) and protease type XXIV (5 mg/ml). Thereafter, the Ca^2+^ concentration was gradually increased to 0.2 mmol/l. Isolation of single cardiomyocytes was conducted in a protease-free solution.

For patch-clamp recordings, pipettes were drawn from borosilicate glass and filled with an intracellular solution containing 6 mM NaCl, 134 mM potassium glutamate, 1 mM NaATP, 1.2 mM MgCl2, and 10 mM HEPES, with pH adjusted to 7.2. The extracellular medium (137 mM NaCl, 5.4 mM KCl, 2 mM CaCl2, 1 mM MgCl2, 1 mM MgSO4, 10 mM Glucose, 5 mM HEPES; pH adjusted to 7.3) was maintained at a temperature of 22°C–23°C. Under current-clamp conditions, action potentials were induced by applying a brief current pulse (2 ms, 1 nA) at a frequency of 0.2 Hz, with a holding current of 40 pA.

To measure potassium currents, the patch pipette was filled with an intracellular solution containing 60 mM KCl, 65 mM potassium glutamate, 3 mM K2ATP, 0.2 mM Na2GTP, 2 mM MgCl2, 5 mM EGTA, and 5 mM HEPES (pH adjusted to 7.2). The cells were continuously bathed in an extracellular solution (5.4 mM KCl, 1 mM CaCl2, 140 mM NaCl, 0.33 mM NaH2PO4, 1 mM MgCl2, 10 mM Glucose, and 5 mM HEPES, with pH adjusted to 7.4). Background potassium currents were recorded from a holding potential of -50 mV by applying incremental voltage steps from -60 mV to +60 mV in 10 mV intervals. After application of the high-affinity TASK-1 inhibitor ‘A293’ (2-(butylsulfonylamino)-N-[(1R)-1-(6-methoxy-3-pyridyl)propyl]-benzamide in DMSO), TASK-1 currents were isolated from the outward potassium currents. Data acquisition and analysis were performed using pCLAMP 10.

### Statistical analysis

IBM SPSS STATISTICS 21 and Microsoft Office Excel software were used to analyze the data. Outliers in the data were determined via IBM SPSS STATISTICS 21 and excluded from further analysis. All data were checked for normal distribution via the Kolmogorov-Smirnov and Shapiro-Wilk test. If appropriate, two-way ANOVA was performed using litter as a cofactor.

## Results

### *Magel2*-KO mice exhibit concentric cardiac remodeling

**Figure 1:**
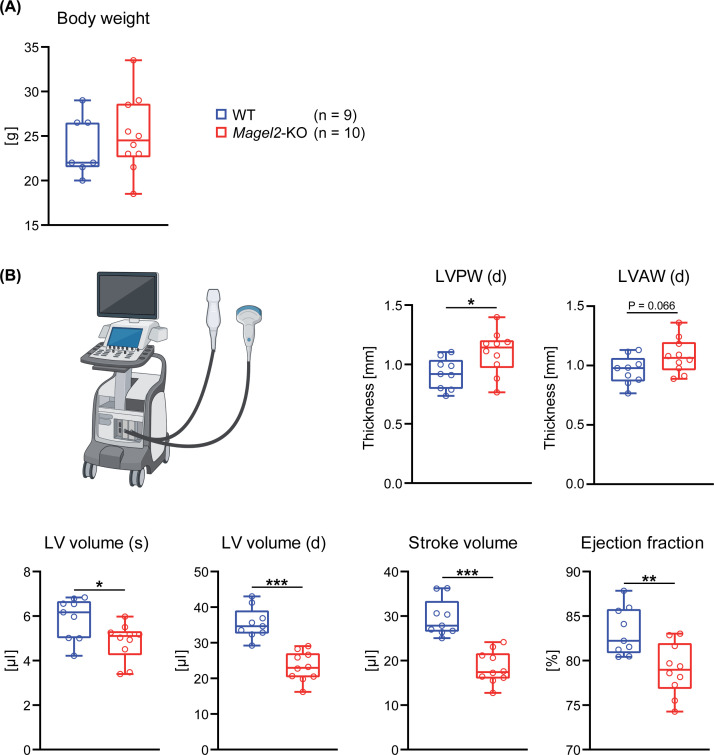
*Magel2*-KO mice exhibit structural and functional alterations of the left ventricle Cardiac structure and function were assessed by transthoracic echocardiography in 20-week-old WT and *Magel2*-KO mice. (**A**) Body weight did not differ between genotypes. (**B**) *Magel2*-KO mice displayed a significant increase in left ventricular posterior wall thickness in diastole (LVPW(d)). Left ventricular anterior wall thickness in diastole (LVAW(d)) did not differ significantly between genotypes (*P* = 0.066). In addition, *Magel2*-KO mice showed reduced left ventricular volumes in systole and diastole, as well as reduced stroke volume and ejection fraction compared to WT littermates. LV, left ventricle; LVPW(d), left ventricular posterior wall thickness in diastole; LVAW(d), left ventricular anterior wall thickness in diastole; (s), systole; (d), diastole. Data are presented as box-and-whisker plots, with boxes representing the interquartile range, whiskers indicating the 95% confidence interval, and the central line representing the median. Statistical significance is indicated by asterisks (*P≤0.05, **P≤0.01, ***P≤0.001; two-sided *t*-test). Figure created using BioRender.com.

To assess cardiac structure and function in male and female *Magel2*-KO mice (defined as animals lacking functional paternal *Magel2* expression), transthoracic echocardiography was performed in 20-week-old WT and *Magel2*-KO animals of both sexes. Because Magel2 is expressed only at very low levels in cardiac tissue, validation of the KO cannot be reliably performed in the heart. We therefore assessed *Magel2* mRNA expression in brain regions with robust physiological expression, including hypothalamus, where Magel2 expression is highest. *Magel2* transcript was undetectable in hypothalamus, cortex and striatum from *Magel2*-KO mice, whereas expression was readily detectable in WT animals (Supplementary Figure S1). Body weight did not differ between genotypes (Figure 1A). In contrast, *Magel2*-KO mice exhibited structural and functional alterations of the left ventricle.

Specifically, left ventricular posterior wall thickness was significantly increased during diastole (approximately 19%), whereas left ventricular volumes were significantly reduced during systole (-18%) and diastole (-34%) (Figure 1B). In line with these structural changes, *Magel2*-KO mice showed a marked reduction in stroke volume (-38%) and a modest but statistically significant decrease in ejection fraction (-5%) (Figure 1B).

Collectively, these findings indicate concentric structural remodeling of the left ventricle.

### *Magel2*-KO mice display a sex-dependent proarrhythmic substrate

**Figure 2:**
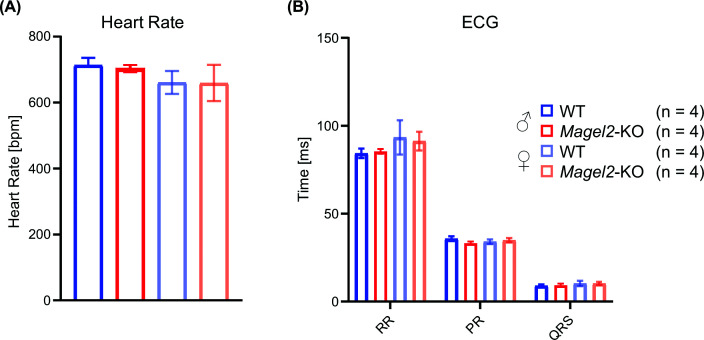
Non-invasive ecgTUNNEL analysis reveals no differences between WT and *Magel2*-KO mice To characterize cardiac electrical activity, male and female WT and *Magel2*-KO mice were subjected to non-invasive tunnel ECG. (**A**) Heart rate. (**B**) RR interval, PR interval, and QRS duration. No differences were detected between WT and *Magel2*-KO littermates. Data are presented as mean ± SD. Statistical analysis was performed using a two-sided *t*-test.

**Figure 3:**
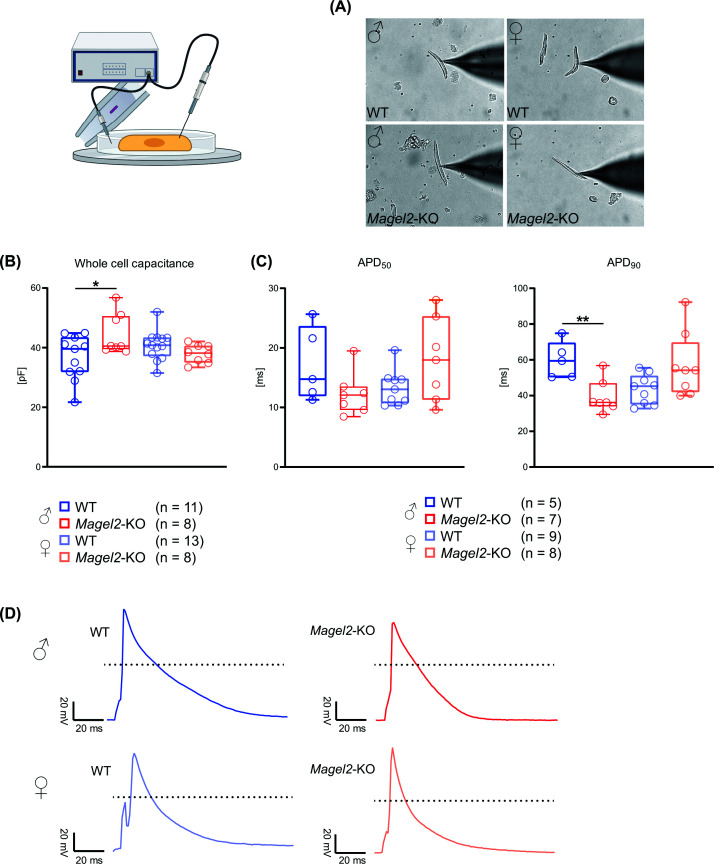
Sex-dependent electrophysiological alterations in atrial cardiomyocytes from *Magel2*-KO mice Electrophysiological properties of isolated atrial cardiomyocytes from male and female WT and *Magel2*-KO mice were analyzed using whole-cell patch-clamp recordings. (**A**) Representative bright-field images of atrial cardiomyocytes obtained from male and female WT and *Magel2*-KO mice during patch-clamp recordings. (**B**) Whole-cell capacitance. (**C**) Action potential duration at 50% (APD_50_) and 90% repolarization (APD_90_). APD_90_ is shortened in atrial cardiomyocytes from male *Magel2*-KO mice compared to male WT cells, whereas no differences were detected in females. (**D**) Representative action potential traces from male and female WT and *Magel2*-KO atrial cardiomyocytes. Data in panel (B) and (C) are shown as box-and-whisker plots; boxes represent the first and third quartiles; whiskers indicate the minimum and maximum values, and the line within the box represents the median. Asterisks denote statistically significant differences (*P≤ 0.05, **P≤0.01; two-sided *t*-test). Figure created using BioRender.com.

To assess potential arrhythmogenic alterations in Magel2-deficient animals, mice of both sexes and genotypes were subjected to non-invasive TUNNEL-ECG monitoring. Analysis showed no significant differences in heart rate (Figure 2A) or ECG intervals, including RR, PR, and QRS duration (Figure 2B), between *Magel2*-KO mice and their littermates. Visual inspection of the ECG recordings confirmed sinus rhythm without evidence of AF or conduction abnormalities during the monitoring period. To further assess potential subtle alterations in cardiac rhythm, heart rate variability was analyzed based on RR intervals derived from the ECG recordings. Representative traces illustrating the relative change of consecutive RR intervals did not reveal evidence of ectopic beats or irregular rhythm. In addition, analysis of RR interval variability based on randomly selected intervals from multiple mice per group (*n* = 3–4 mice per group) showed no differences between WT and *Magel2*-KO animals, consistent with a regular sinus rhythm (Supplementary Figure S2). At first glance, this suggests the absence of arrhythmias, atrioventricular blocks, or repolarization abnormalities. However, patch-clamp analyses of isolated atrial cardiomyocytes from male animals revealed significant differences compared to WT cells. These changes were not observed in female animals (Figure 3). Specifically, male *Magel2*-KO atrial cardiomyocytes exhibited a significantly increased whole cell capacity, reflecting increased cell size (Figure 3B), and a shortened action potential duration at 90% repolarization (APD_90_), reduced by about 34% compared to male WT cells (Figure 3C,D).

**Figure 4:**
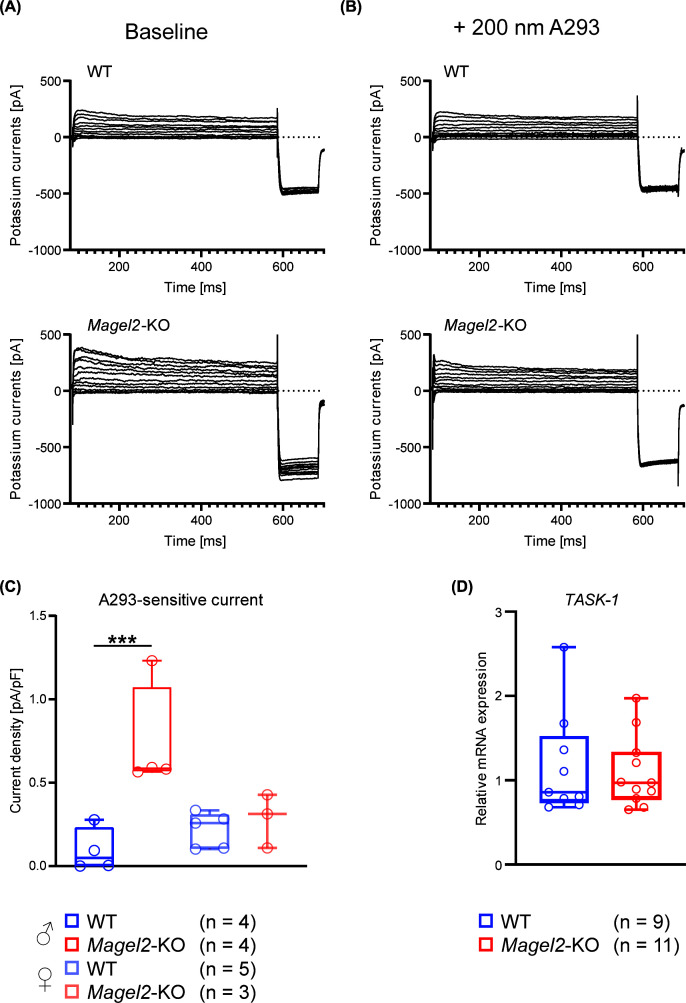
Increased TASK-1-mediated potassium currents in atrial cardiomyocytes from male *Magel2*-KO mice Potassium currents were recorded from isolated atrial cardiomyocytes of male and female WT and *Magel2*-KO mice using whole-cell patch-clamp recordings. (**A**) Representative baseline potassium current traces from WT and *Magel2*-KO cardiomyocytes. (**B**) Potassium current traces recorded in the presence of the TASK-1 inhibitor A293 (200 nM). (**C**) A293-sensitive potassium current density. TASK-1-specific current density is increased in cardiomyocytes from male *Magel2*-KO mice compared to male WT cells, whereas no differences were detected in females. (**D**) Relative *TASK-1* (*Kcnk3*) mRNA expression in cardiac tissue from WT and *Magel2*-KO mice. Data are presented as box-and-whisker plots; boxes represent the first and third quartiles; whiskers indicate the minimum and maximum values, and the line within the box represents the median. Numbers of analyzed cells or animals are indicated in the figure. Asterisks denote statistically significant differences (***P≤0.001; two-sided *t*-test).

Moreover, an elevated baseline potassium current was detected in male *Magel2*-KO cardiomyocytes (Figure 4A). This current was significantly reduced by inhibition of the TASK-1 potassium channel (TWIK-related acid-sensitive K^+^ channel; K2P3.1) using the experimental ion channel inhibitor A293 (Figure 4B).

The TASK-1-specific current density, determined by A293 inhibition, was increased by about 690% in cardiomyocytes of male *Magel2*-KO mice compared to those of male WT mice. Again, no differences between the genotypes were detected in females (Figure 4C). These data indicate increased functional TASK-1 current density in male *Magel2*-KO animals.

An analysis of cardiac *TASK-1* mRNA expression, however, revealed no statistically significant difference between the genotypes; based on the observed variability and sample size, the analysis was limited to resolving relatively large expression differences of approximately 59% or greater (Figure 4D).

### Aged *Magel2*-KO mice exhibit impaired glycemic control

**Figure 5:**
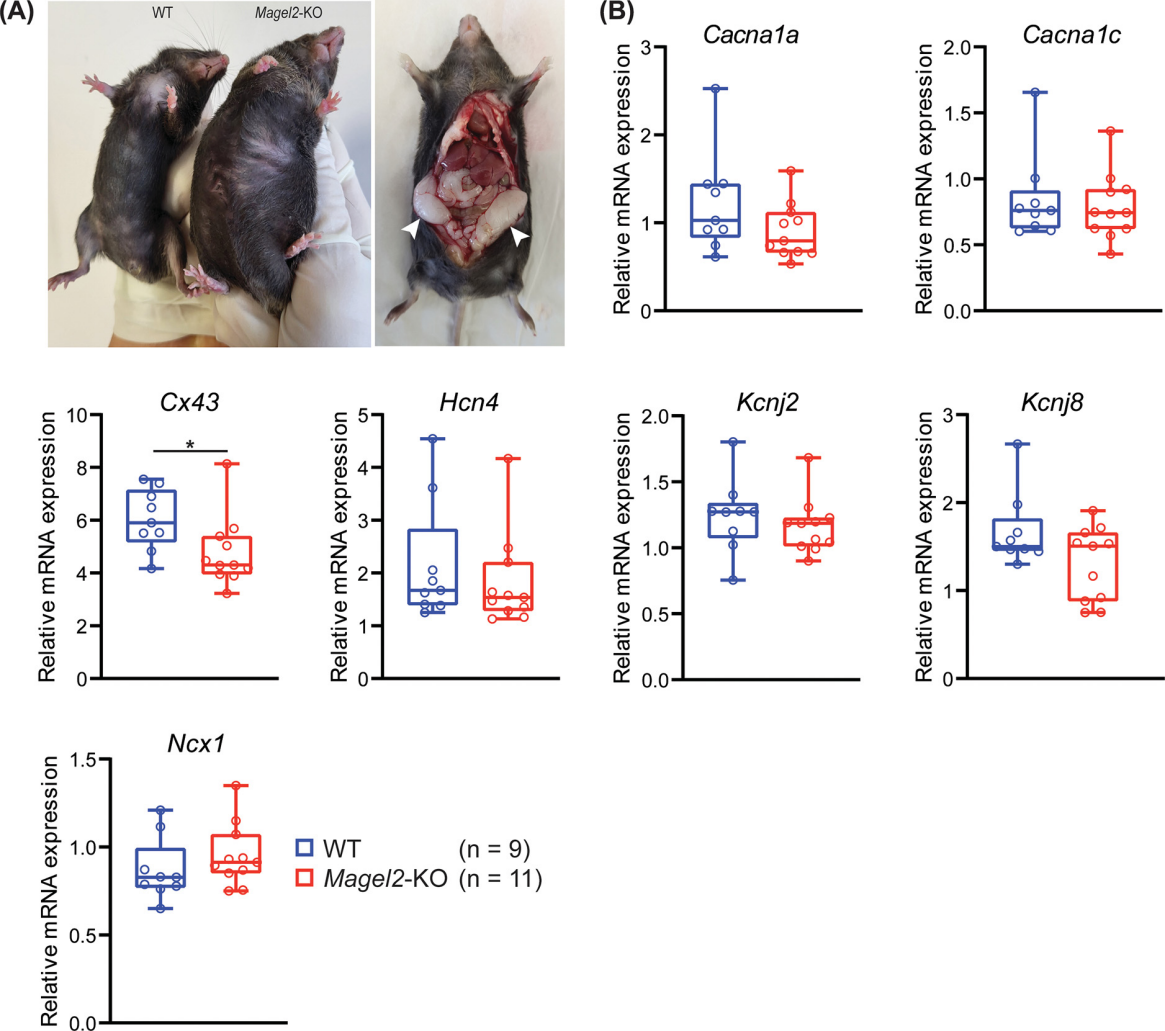
Reduced cardiac *Cx43* mRNA expression in aged *Magel2*-KO mice (**A**) Representative images of aged WT and *Magel2*-KO mice illustrating increased adiposity in *Magel2*-KO animals. (**B**) Relative mRNA expression levels of cardiac ion channels, ion transporters, and gap junction proteins (*Cacna1a, Cacna1c, Cx43, Hcn4, Kcnj2, Kcnj8*, and *Ncx1*) in hearts from aged WT and *Magel2*-KO mice (>51 weeks). Cx43 mRNA expression is reduced in *Magel2*-KO hearts, whereas no differences were detected for the other analyzed genes. Data are presented as box-and-whisker plots; boxes represent the first and third quartiles; whiskers indicate the minimum and maximum values, and the line within the box represents the median. Asterisks denote statistically significant differences (*P≤0.05; two-sided *t*-test).

To explore potential mechanisms underlying the observed structural cardiac remodeling and electrophysiological alterations in *Magel2*-KO mice, further analyses were performed. As adult *Magel2*-KO animals develop pronounced obesity with increasing age [14] (Figure 5A), which may promote metabolic disturbances and cardiovascular dysfunction, mice older than 51 weeks were examined.

Given that metabolic alterations can influence the expression of cardiac ion channels, ion transporters, and gap junction proteins, we analyzed the mRNA expression levels of seven genes (*Cacna1a, Cacna1c, Cx43, Hcn4, Kcnj2, Kcnj8*, and *Ncx1*) that are frequently associated with heart failure and/or arrhythmia.

**Figure 6:**
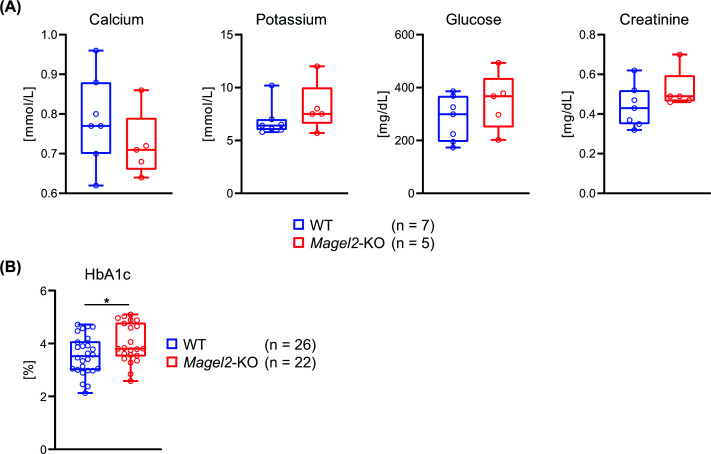
Increased HbA1c levels in aged *Magel2*-KO mice (**A**) Serum electrolyte concentrations, kidney function parameters, and random blood glucose levels in aged WT and *Magel2*-KO mice. No differences were detected between genotypes. (**B**) Glycated hemoglobin (HbA1c) levels in aged WT and *Magel2*-KO mice. HbA1c is increased in *Magel2*-KO animals compared to WT controls. Data are presented as box-and-whisker plots; boxes represent the first and third quartiles; whiskers indicate the minimum and maximum values, and the line within the box represents the median. Asterisks denote statistically significant differences (*P≤0.05; two-sided *t*-test).

This analysis revealed a significant reduction in *Cx43* mRNA expression by approximately 21% in *Magel2*-KO hearts, whereas no statistically significant differences were observed for the other examined genes (Figure 5B). Based on the observed variability and sample size, the sensitivity of these analyses differed between targets, and smaller expression differences could not be excluded for several genes. Blood analyses showed no statistically significant differences in serum electrolyte concentrations, kidney function parameters, or random blood glucose levels between *Magel2*-KO and WT mice (Figure 6A). Based on the observed variability and sample size, however, the glucose analysis was limited to resolving relatively large differences of approximately 61% or greater.

To assess long-term glycaemic control, glycated hemoglobin (HbA1c) levels were determined. *Magel2*-KO mice exhibited a 13% increase in HbA1c compared to WT animals (Figure 6B), consistent with mildly impaired long-term glycaemic control rather than overt diabetes.

## Discussion

Muscular hypotonia, delayed motor development, and impaired mobility are relevant clinical features of PWS and SYS [3,4,9]. Magel2-deficient mice, a model relevant to PWS and SYS, exhibit reduced strength and endurance. These deficits may in part be attributable to functional impairments of skeletal muscle, as *Magel2*-KO animals display mild structural alterations of muscle fibers [14]. However, reduced exercise tolerance is also a hallmark of cardiovascular disease [15,16]. Importantly, cardiovascular disease represents one of the leading causes of mortality in individuals with PWS and was identified as the second most frequent cause of death in a large 40-year mortality survey [7]. In addition, autonomic dysfunction and structural cardiac abnormalities have been reported in pediatric patients with PWS. Although MAGEL2 is expressed only at very low levels in human cardiac tissue [17], cardiac involvement has been documented in children with PWS [18], and case reports describe functional cardiac abnormalities in individuals with SYS [9,10]. In line with these observations, the present study demonstrates echocardiographically detectable structural and functional cardiac alterations together with electrophysiological changes in cardiomyocytes derived from *Magel2*-KO mice.

Magel2-deficient animals exhibited reduced left ventricular end-diastolic volume and stroke volume, together with structural changes consistent with concentric remodeling. Ejection fraction was modestly but significantly reduced compared to WT mice. An important limitation of the present study is that Magel2 deficiency alone does not fully recapitulate the genetic complexity of PWS, which involves loss of multiple paternally expressed genes within the 15q11–q13 locus including *SNORD116*. Similarly, SYS is typically caused by truncating mutations in *MAGEL2*, which may differ functionally from complete loss of gene expression. Therefore, the *Magel2*-KO model reflects one defined molecular component contributing to disease biology relevant to PWS and SYS rather than the full clinical spectrum of these disorders. Nevertheless, investigation of Magel2 deficiency provides mechanistic insight into biological processes that may contribute to cardiovascular alterations observed in affected individuals.

According to current American and European heart failure guidelines, heart failure with preserved ejection fraction (HFpEF) is defined by preserved ejection fraction in combination with symptoms of heart failure and objective evidence of diastolic dysfunction [19,20]. Although formal echocardiographic indices of diastolic dysfunction, such as transmitral E/A or E/E′ ratios, were not assessed in the present study and the diagnostic criteria for HFpEF are therefore not fulfilled, the remodeling pattern observed in *Magel2*-KO mice shares certain structural characteristics that are also encountered in clinical conditions with preserved systolic function [21,22]. Further characterization of diastolic filling dynamics would be required to determine whether intrinsic diastolic dysfunction is present in this model.

As the clinical severity of heart failure is closely linked to exercise intolerance and fatigue, functional performance represents an important translational correlate [15]. Consistent with this, *Magel2*-KO animals demonstrate reduced exercise capacity in treadmill testing compared to WT littermates [14].

AF commonly occurs in the setting of structural and functional cardiac remodeling, and atrial myopathy is thought to promote both the development and persistence of AF [23]. Conversely, AF may further aggravate cardiac remodeling and functional impairment [24]. Although surface ECG recordings did not reveal AF in *Magel2*-KO mice, the shortened action potential duration at 90% repolarization observed in cardiomyocytes from these animals indicates increased arrhythmogenic susceptibility, as abbreviated repolarization and refractory periods facilitate the formation of reentry circuits. Increased functional activity of the outwardly rectifying potassium channel TASK-1 has been reported in the context of chronic AF [25]. As TASK-1 channels are predominantly expressed in atrial tissue, electrophysiological analyses in the present study focused on atrial cardiomyocytes. Specific inhibition of TASK-1 normalized the elevated potassium currents in *Magel2*-KO cardiomyocytes, suggesting that TASK-1 contributes to the observed shortening of APD90. However, *TASK-1* mRNA expression was not increased in *Magel2*-KO hearts, indicating that mechanisms other than transcriptional upregulation may underlie the enhanced potassium currents. In addition, cardiac *Cx43* mRNA expression was significantly reduced in *Magel2*-KO hearts. As altered Cx43 expression has been implicated in both AF and forms of cardiac remodeling associated with preserved ejection fraction, connexins represent relevant targets for future studies aimed at further elucidating the electrophysiological substrate underlying the observed phenotype [26,27].

Notably, the observed electrophysiological alterations were predominantly detected in male *Magel2*-KO mice, suggesting potential sex-dependent differences in cardiac ion channel regulation that warrant further investigation.

Assessment of arrhythmia susceptibility in SYS is of particular importance, as AF frequently occurs asymptomatically or subclinically. Undiagnosed or subclinical AF significantly increases the risk of left atrial thrombus formation and subsequent cardioembolic ischemic stroke [28,29].

Both AF and heart failure with preserved systolic function are closely linked to metabolic dysregulation [22,30]. Insulin resistance represents a central component of the metabolic syndrome and is strongly associated with the development of both AF and heart failure with preserved ejection fraction [22,31,32]. Importantly, individuals with PWS frequently develop obesity, insulin resistance, and type 2 diabetes, and metabolic disturbances have also been described in individuals with SYS, although systematic data in this population remain limited [33,34]. Although random serum glucose levels did not differ between genotypes, aged *Magel2*-KO animals exhibited a significantly increased HbA1c fraction. Food intake was not assessed in the present cohort, and we therefore cannot determine whether impaired glycaemic control occurred independently of caloric intake. However, this question has previously been examined in the same *Magel2*-KO mouse model. Bischof et al. demonstrated post-weaning weight gain, increased adiposity, and altered metabolic parameters in Magel2-deficient mice, while Knani et al. further investigated obesity-related mechanisms in this model in the context of therapeutic modulation of the endocannabinoid system [35,36]. Together, these studies indicate that Magel2 deficiency promotes metabolic alterations relevant to obesity and glucose homeostasis. In addition, studies of the Snord116-deficient mouse model illustrate that different genes within the PWS locus contribute distinct components of the metabolic phenotype [37]. As HbA1c reflects average blood glucose levels over the preceding 8–12 weeks and serves as an established diagnostic marker for diabetes mellitus, this finding indicates chronically impaired glycemic control in *Magel2*-KO mice. The observed difference in HbA1c was moderate in magnitude and is therefore best interpreted as evidence of mildly impaired long-term glycaemic control rather than overt diabetes. In this context, the lack of a significant difference in random blood glucose is not unexpected, as single time-point glucose measurements are less sensitive than HbA1c for detecting chronic, low-grade disturbances in glucose homeostasis.

In addition, the sample size for the remaining metabolic parameters was smaller than for HbA1c, and the glucose analysis was limited to resolving relatively large differences; therefore, more subtle differences in these measures cannot be excluded. Given the altered body composition of these animals, metabolic dysregulation is likely to contribute to the development of the observed cardiac abnormalities. Chronic low-grade metabolic stress and impaired glycaemic control are known to promote myocardial remodeling and electrical instability, providing a plausible mechanistic link between the metabolic and cardiac phenotype observed in *Magel2*-KO mice. In light of the low expression of Magel2 in cardiac tissue, the observed cardiac phenotype is therefore more likely influenced by systemic and neuroendocrine mechanisms rather than direct cardiomyocyte-autonomous effects.

The demonstration of structural cardiac remodeling and electrophysiological alterations consistent with increased arrhythmogenic susceptibility in a Magel2-deficient mouse model has important implications for individuals with SYS and PWS. In conjunction with the high prevalence of sleep apnea and other cardiovascular risk factors, these findings suggest that individuals with SYS and PWS may be at increased risk of cardiac complications, including arrhythmias and adverse cardiac remodeling. Comprehensive cardiovascular evaluation, including screening for AF or other proarrhythmogenic alterations, may therefore represent a valuable component of clinical management in these patient populations.

## Clinical perspectives

- PWS and SYS are primarily regarded as neurodevelopmental disorders; however, cardiovascular morbidity represents a major cause of mortality in PWS, and cardiac abnormalities have been reported in SYS, suggesting that MAGEL2 deficiency may have systemic effects beyond the central nervous system.
- Using *Magel2*-KO mice—an established model of MAGEL2 deficiency relevant to both SYS and PWS—we identify structural and functional cardiac alterations characterized by reduced left ventricular filling volumes, concentric remodeling, preserved systolic function, and electrophysiological changes consistent with increased arrhythmogenic susceptibility, together with evidence of impaired glycemic control in aged animals.
- These findings expand the phenotypic spectrum of MAGEL2 deficiency to include clinically relevant cardiac involvement and support the consideration of structured cardiovascular surveillance and metabolic risk assessment in individuals with PWS and SYS.

## Supplementary Material

Supplementary Figure S1-S2 and Table S1

## Data Availability

All data supporting the findings of this study are included within the article and its supplementary information files. No additional datasets requiring deposition in public repositories were generated during this study.

## Open Access

Open access for this article was enabled by the participation of Heidelberg University in an all-inclusive Read & Publish agreement with Portland Press and the Biochemical Society under an agreement with the TIB German Alliance.

## Abbreviations

AF: atrial fibrillation
ECG: electrocardiography
KO: knockout
PWS: Prader–Willi syndrome
SYS: Schaaf–Yang syndrome
WT: wild-type
