# Supplementary material for: Magel2 deficiency promotes cardiac remodeling and increases arrhythmogenic susceptibility in a mouse model relevant to Prader–Willi and Schaaf–Yang syndromes
Source: Clin Sci (Lond). 2026 Jun 22;140(7):1415–27. doi: 10.1042/CS20260647 (PMC13286937; doi:10.1042/CS20260647)
Supplement: Supplementary Figure S1-S2 and Table S1 [file CS-2026-0647_supp.pdf]

Suppl. Fig. 1

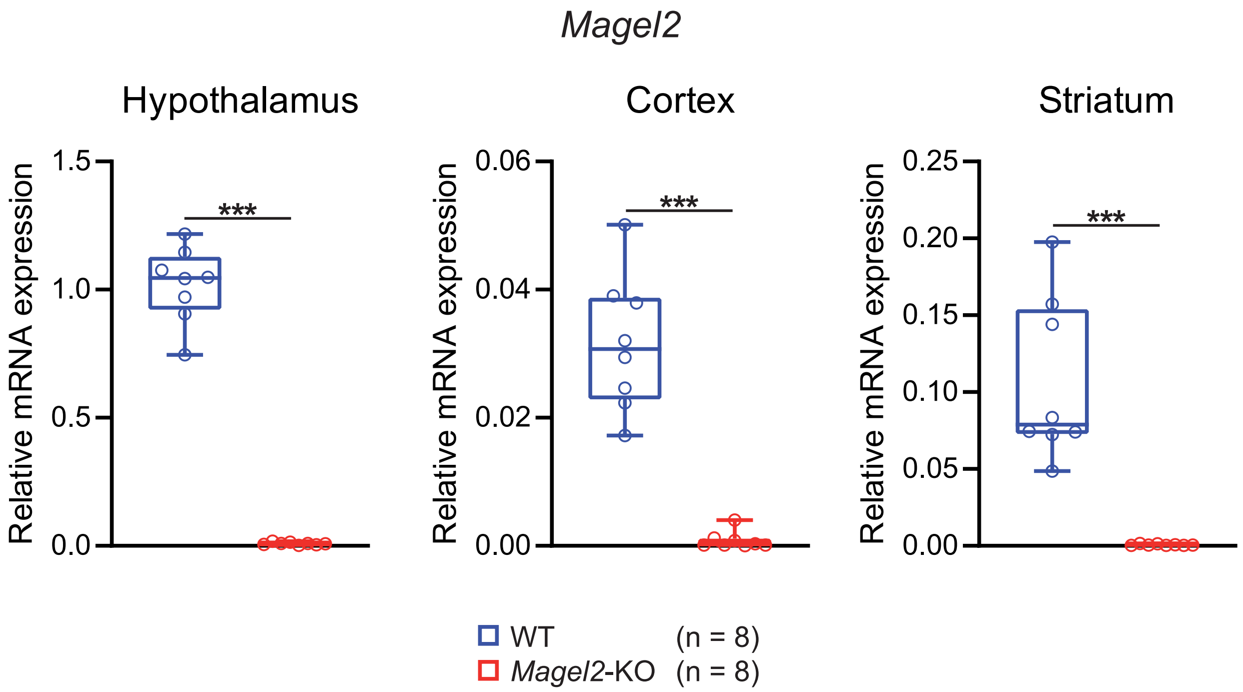

Supplementary Figure 1. **Validation of *Magel2* transcript loss in *Magel2*-KO mice.** Relative *Magel2* mRNA expression in hypothalamus, cortex and striatum from adult WT and *Magel2*-KO mice, determined by quantitative real-time PCR. *Magel2* transcript was undetectable in *Magel2*-KO animals in all three analysed brain regions, confirming effective disruption of Magel2 expression. Asterisks denote statistically significant differences (***P ≤ 0.001; two-sided t-test).

Suppl. Fig. 2

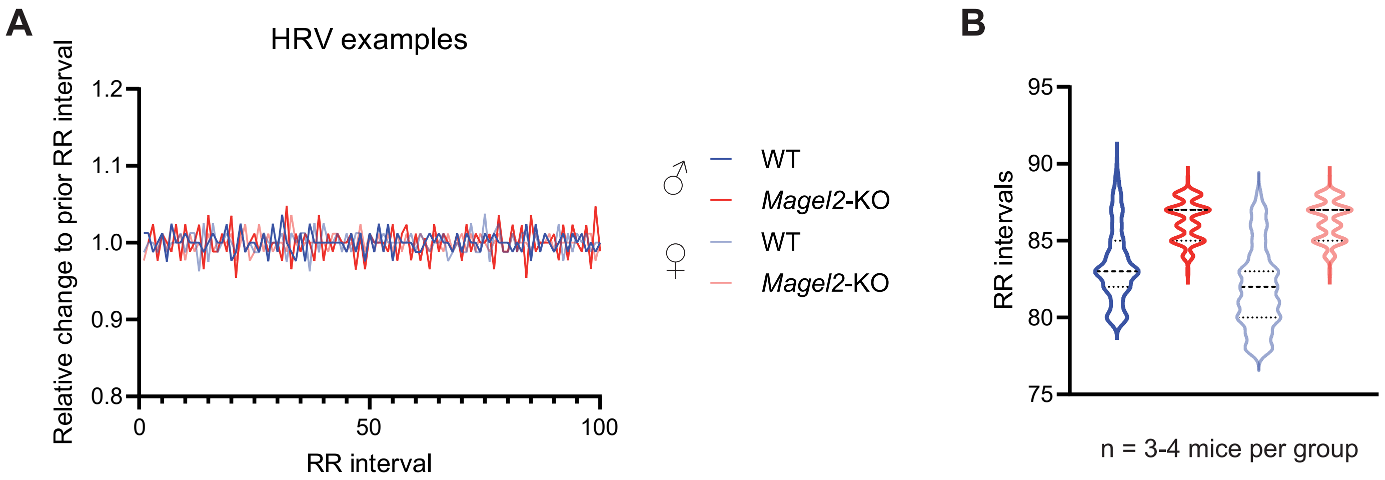

Supplementary Figure 2. **Heart rate variability analysis in WT and *Magel2*-KO mice. A**, Representative traces showing the relative change of consecutive RR intervals from one mouse per group (100 consecutive beats). No evidence of ectopic beats or irregular rhythm was observed. **B**, Analysis of RR interval variability based on 200 RR intervals randomly selected from multiple mice per group (n = 3-4 mice per group). No differences in RR interval variability were detected between WT and *Magel2*-KO mice, consistent with a regular sinus rhythm.

Suppl. Table 1

**Primer**	**Sequence 5'-3'**
Cacna1a for	CTGACATCGCGTCTGTGG
Cacna1a rev	TTCTCCAGCTTGGCACTTTT
Cacna1c for	GTGCAAGACACAGCCAATAAAG
Cacna1c rev	CGGTTGAAGAGGGACACAAA
Cx43 for	TCCTTTGACTTCAGCCTCCA
Cx43 rev	CCATGTCTGGGCACCTCT
Hcn4 for	GACAGCGCATCCATGACTAC
Hcn4 rev	ACAAAGTTGGGATCTGCGTT
Hprt1 for	TCCTCCTCAGACCGCTTT
Hprt1 rev	CCTGGTTCATCCATCGCTAATC
Kcnj2 for	TGAAGTTGCCCTAACAAGCA
Kcnj2 rev	GCTCTCTGGGACTCCGTTCT
Kcnj8 for	GAGAAAGGCACCATGGAGAA
Kcnj8 rev	GGAGAAGAGAAACGCAGACG
Ncx1 for	CCATCCTAGGCGAGCACA
Ncx1 rev	TCGTCTTCTTAATGAGTTTGTCCA
Sdha1 for	CATGCCAGGGAAGATTACAAA
Sdha1 rev	GTTCCCCAAACGGCTTCT
TASK1 for	CAACGTCTATGCCGAGGTGC
TASK1 rev	CGGGATGATCATGGGGATGG
Magel2 for	GGGCATTCCTCGAAACCAAC
Magel2 rev	CATGGGACTCTTCGGCGG

Supplementary Table 1. **Primer sequences used for quantitative real-time PCR.**
